# Relationships between state-level general population alcohol policies and birth outcomes: Results from 1972–2019 vital statistics

**DOI:** 10.1371/journal.pone.0327559

**Published:** 2025-08-04

**Authors:** Meenakshi S. Subbaraman, Nancy F. Berglas, William C. Kerr, Sarah C. M. Roberts

**Affiliations:** 1 Behavioral Health and Recovery Studies, Public Health Institute, Oakland, California, United States of America; 2 Advancing New Standards in Reproductive Health (ANSIRH), Bixby Center for Global Reproductive Health, University of California, San Francisco, Department of Obstetrics, Gynecology & Reproductive Sciences, Oakland, California, United States of America; 3 Alcohol Research Group, Public Health Institute, Emeryville, California, United States of America; Christian Medical College, INDIA

## Abstract

**Background:**

Research has found that policies that single out pregnant people’s alcohol consumption are mostly ineffective. Identifying alternative approaches – for example, general population alcohol policies – that can reduce adverse effects of pregnant people’s alcohol consumption is essential. Here, we examine how U.S. state-level alcohol policies regarding grocery store and gas station sales, Sunday sales, Blood Alcohol Concentration limits for driving, and government monopolies relate to birth outcomes.

**Methods:**

Outcome data came from the 1972–2019 U.S. Vital Statistics System birth certificates (N = 160,538,939 live singleton births). Primary outcomes were low birthweight (<2,500 grams) and preterm birth (<37 weeks). Fully adjusted models included state and year fixed effects, state-specific time trends, and maternal- and state-level covariates with standard errors clustered by state.

**Results:**

The only significant, robust associations between policies and outcomes were for government monopolies. In fully adjusted models, having a government monopoly on spirits or on both spirits and wine retail sales (vs. none) were each related to lower odds of low birthweight births (aOR=0.94, 95% CI: 0.93, 0.95; aOR=0.95, 95% CI: 0.92, 0.98 respectively). Having a government monopoly on spirits sales was also significantly related to lower odds of preterm births (aOR=0.97, 95% CI: 0.95, 1.00).

**Conclusions:**

Government monopolies on spirits and wine relate to better birth outcomes. Findings underscore the importance of maintaining state government monopolies on spirits and wine as a strategy for protecting against adverse effects of pregnant people’s drinking.

## Introduction

### Alcohol use during pregnancy

Nearly one in seven pregnant people in the U.S. reported current drinking from 2018–2020, with 5.2% reporting binge drinking in the past 30 days [1]. Drinking at even low levels is linked to adverse birth outcomes like preterm birth and low birthweight [2]. As one approach to reduce pregnant people’s alcohol consumption and related harms, U.S. states have adopted various policies aimed at reducing alcohol use among pregnant people. For example, 25 states mandate that signs warning of adverse effects of consuming alcohol during pregnancy be posted in places that sell alcohol [3]. However, a growing number of studies show that policies specifically focused on alcohol use among pregnant people are ineffective [4–7]. One study of U.S. births from 1972–2013 found that out of eight policies regarding alcohol use during pregnancy, five were significantly related to higher odds of low birthweight and/or preterm birth [4]. These same policies are generally not associated with decreased infant morbidities or maltreatment, and, in some cases, associated with increased infant morbidities and maltreatment [6].

Various mechanisms could explain why pregnancy-specific policies are ineffective. For example, pregnant people who use alcohol and/or drugs might fear that getting prenatal care or treatment will lead to child protective services reporting and child removal, and some may avoid prenatal care out of a belief that their substance use has already irreversibly damaged their fetus [8–10]. Supporting this, child abuse/neglect policies and policies mandating posting of mandatory warning signs for alcohol use during pregnancy are associated with less prenatal care utilization [4]. Furthermore, recent survey and qualitative research also finds that people who use cannabis during pregnancy do not trust the information in warning signs [11,12]. Similar mechanisms could be at work for mandatory warning signs for alcohol. Given the lack of protective associations found for pregnancy-specific alcohol policies and the evidence for reasons why these policies might not work, recommendations for future work include consideration of broader alcohol policies that are not specific to pregnant people [6,13].

### General population alcohol policies and outcomes related to drinking during pregnancy

Despite a call almost 25 years ago for researchers and policymakers to consider that general population alcohol policies might be relevant for reducing harms from pregnant people’s drinking [14], until recently, few studies have examined how general population alcohol policies relate to outcomes associated with pregnant people’s drinking. Studies of alcohol taxes show that increased taxes are related to lower incidence of preterm and low birthweight births [15,16], child maltreatment [17,18], and alcohol-related infant and maternal morbidities [19]. Fewer studies have examined how outcomes associated with pregnant people’s drinking might be impacted by alcohol availability policies, which restrict the times, types of places, and number of places where alcohol can be sold. A Canadian study on alcohol availability found that more alcohol outlets within 30 minutes of home was related to more drinking during pregnancy but not related to birth outcomes [20]. Similarly, a U.S. study of women of reproductive age found that prohibiting Sunday off-premise spirits sales, government monopolies on spirits sales, restrictions on beer sales at gas stations, and stricter Blood Alcohol Content (BAC) limits for driving were all related to less past 12-month drinking [21]. As people do not typically start drinking upon becoming pregnant, policies that reduce drinking among women of reproductive age should be relevant for reducing adverse effects of drinking during pregnancy. Supporting this, analyses examining U.S. state alcohol availability policies found that government monopolies and restrictions on gas station spirits sales were each associated with reduced odds of alcohol-related infant morbidities and injuries [22]. However, no study to date has examined whether this range of general population alcohol availability policies in the U.S. relate to improved birth outcomes.

### Study aim

Thus, the aim of this study is to examine how state-level policies regarding grocery and gas station heavy beer and spirits sales, Sunday spirits sales, BAC limits for driving, and government control of spirits and wine retail sales relate to birth outcomes. These general population alcohol policies have the most complete state/year-level data available for the period under study. Alcohol tax data are not available for government monopoly states and are thus excluded from current analyses. Based on prior findings, we hypothesize that stricter alcohol availability policies and stricter BAC limits for driving will be related to fewer low birthweight and preterm births.

## Materials and methods

### Sample

Outcome data came from the 1972–2019 U.S. National Center for Health Statistics Vital Statistics System birth certificates (N = 160,538,939 live singleton births). We excluded multiple births because babies born in multiples are known to be at higher risk for adverse birth outcomes [23]. We also restricted data to births conceived in February 2019 or earlier to avoid any potential confounding due to the onset of the COVID-19 pandemic [24]. From 1972 to 1984, Vital Statistics records include 50–100% of the births in each state; from 1985 to 2019, records include 100% of births for all states. Because gas station policy data were collected starting in 1978, analyses including the gas station policy were restricted to births from 1978–2019 (N = 155,546,474). This secondary analysis study was approved by the University of California, San Francisco’s Institutional Review Board.

### Outcomes

Primary outcomes were low birthweight (<2,500 grams) and preterm birth (<37 weeks). Continuous birthweight and gestational age were obtained from birth certificates and dichotomized for analyses. Outcome data were merged with state/year-level alcohol policies on state of mother’s residence and year of conception.

### State/year-level alcohol policies

We examined five time-varying state-level policies: (1) grocery store sales, (2) gas station sales, (3) Sunday off-premise spirits sales, (4) BAC limits for driving, and (5) government monopoly control over retail sales. Grocery and gas station sales were coded as three categories: (i) spirits and heavy beer permitted [referent]; (ii) spirits sales prohibited (i.e., heavy beer sales only permitted); or (iii) both spirits and heavy beer sales prohibited. Sunday off-premise sales were coded as dichotomous permitted [referent] vs. not. BAC limits for driving were coded as three categories: no law [referent],.10 limit, or.05−.08 limit. Finally, government monopolies were coded as three categories: (i) privatized (i.e., no government monopoly) [referent]; (ii) government monopoly on spirits retail; or (iii) government monopoly on spirits and wine retail.

Data on whether heavy beer (>3.2% alcohol by volume) or spirits sales were permitted at grocery stores or gas stations came from the Spirits Handbooks for 1972–2014 (1978–2014 for gas stations; [25,26]) and the National Alcoholic Beverage Control Association (NABCA) for 2015–2019 [27]. Data for off-premise Sunday spirits sales came from the Distilled Spirits Council of the United States [28] for 1972–2003 and from NABCA for 2004–2019. BAC limit data came from the National Highway Traffic Safety Administration (NHTSA; [29]) and original legal research for 1972–1987, NHTSA and NIAAA’s Alcohol Policy Information System (APIS; [30]) for 1988–1997, and from APIS for 1998–2019. Data for state government monopolies on wine or spirits retail came from the Spirits Handbooks for 1972–1997 [26,31] and from APIS for 1998–2019 [30]. For Sunday sales, 13 states with local variability (e.g., local options) were coded based on the policy for the state’s most populous county.

### Statistical analyses

We used logistic regression to test whether policies are related to odds of low birthweight or preterm birth. For each outcome, the first set of regressions modeled each policy separately and the second set of regressions modeled all policies simultaneously. The two sets of regressions each included (Model 1) unadjusted models; (Model 2) models adjusted for state and year fixed effects; (Model 3) models with state and year fixed effects, further adjusted for individual-level covariates [maternal age (categorical: 15–19, 20–24, 25–29, 30–34, 35–29, 40–44, ≥ 45), race (White, Black, Asian/Pacific Islander, Other/Missing), marital status (married, not married, missing), education (less than high school, high school equivalent, more than high school, missing), parity (no previous live births, one previous live birth, more than one previous live birth, missing)], and state-year level covariates (per capita cigarette consumption, unemployment, and percent living in poverty); and (Model 4) models adjusted for fixed effects, individual and state covariates, and state-specific linear, quadratic, and cubic trends. State-level cigarette consumption was included as a proxy for maternal cigarette use, as tobacco use is inconsistently and poorly measured in birth certificate data. All models clustered standard errors by state.

We also ran four sets of sensitivity analyses. First, we ran Model 4 further adjusting for per capita alcohol consumption; we did not adjust for per capita alcohol consumption in main analyses because it could be on the causal pathway between policy exposures and outcomes, and therefore adjustment for per capita consumption may attenuate estimates. Still, we conducted sensitivity analyses adjusting Model 4 for time-varying state-level per capita alcohol consumption because it could influence state policy adoption. Second, we conducted separate analyses further adjusting for pregnancy-specific policies previously found related to outcomes [4]. These models simultaneously adjusted for separate state-year indicators of policies that (1) mandate warning signs about drinking during pregnancy where alcoholic beverages are sold; (2) address the legal significance of conduct during pregnancy and damage caused in utero, and, in some cases, defines alcohol use during pregnancy as child abuse or neglect; (3) prohibit use of the results of medical tests as evidence in criminal prosecution of pregnant people who may have caused harm to a fetus or child; (4) give pregnant people priority access to substance use treatment. Preterm birth sensitivity analyses additionally adjusted for (5) policies that require reporting alcohol use during pregnancy for assessment/treatment purposes and/or data gathering purposes. Third, given significant changes to birth certificates in 1989 [32], including for recording of gestation [33], we restricted Model 4–1989–2019 (N = 120,119,355 for low birthweight births; N = 119,365,882 for preterm births). Finally, because data for gas stations are only available starting in 1978, we ran simultaneous policy models for the 1972–2019 period without the two gas station policies (N = 160,451,324 for low birthweight births; N = 156,692,194 for preterm births).

## Results

### Descriptives

**Fig 1:**
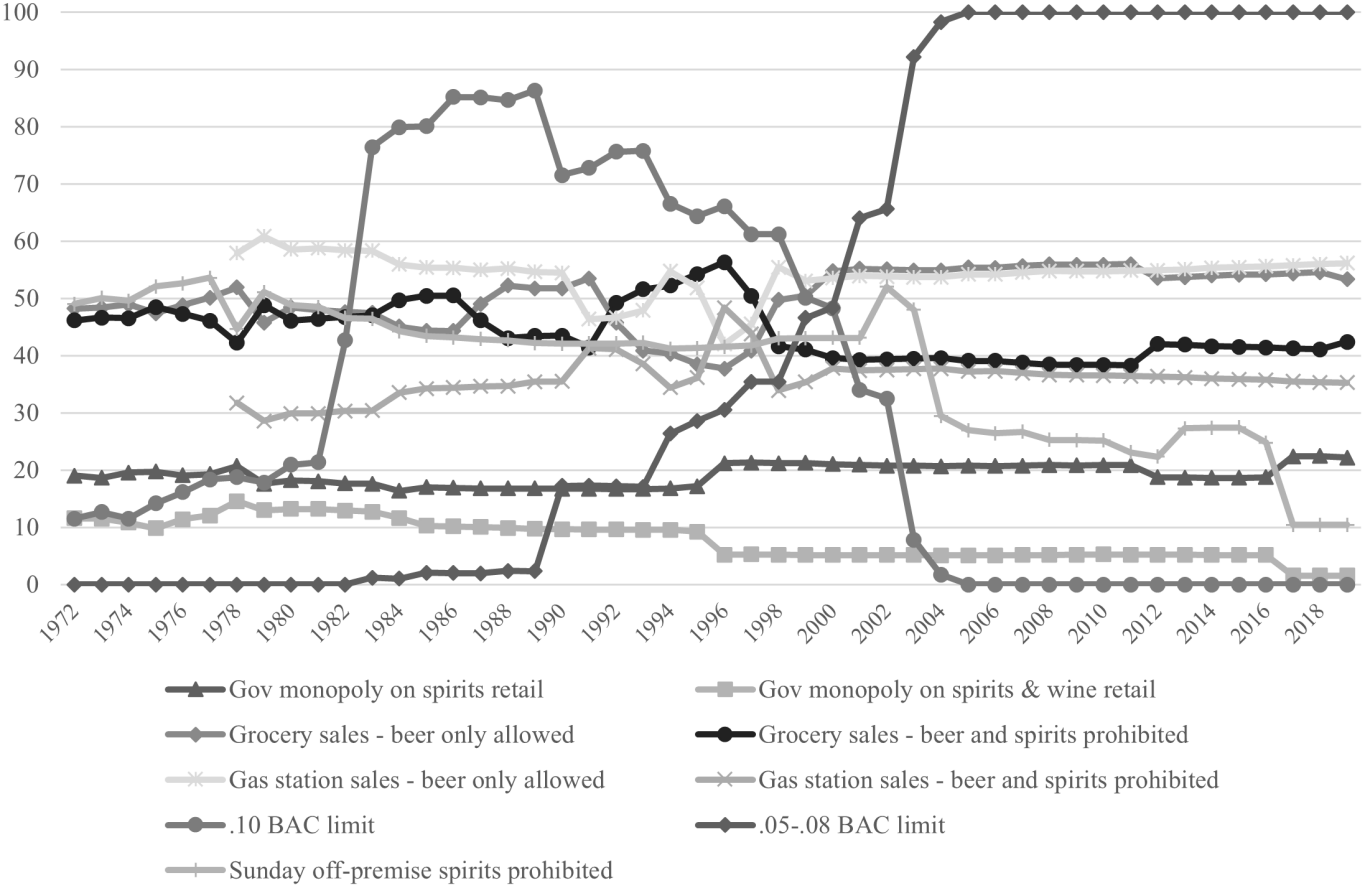
Percentage of births in dataset exposed to each policy, 1972-2019.

Fig 1 shows the proportion of births exposed to each policy from 1972−2019. The proportion of births exposed to each grocery store, gas station, and government monopoly policy appear relatively stable with a low proportion (<25%) exposed to government monopolies on spirits and/or wine retail sales for the entire study period. Conversely, the proportion of births exposed to prohibitions on Sunday off-premise spirits sales fluctuates over the study period, starting at approximately 45% and ending at approximately 10%. The proportion of births exposed to each BAC limit law also varies substantially during the study period, as most states did not have BAC limit laws in 1972 but all had adopted.05−.08 limits by 2005.

### Associations between state-level policies and birth outcomes

Table 1 shows results from models regressing birth outcomes on each alcohol policy separately. For each policy, Model 1 is unadjusted, Model 2 adjusts for state and year fixed effects, Model 3 further adjusts for individual- and state-level covariates, and Model 4 further adjusts for trends. Results are generally consistent across models though more relationships become significant at the *P* < .05 level in adjusted models. We focus on results from fully adjusted Model 4 which show that, in models examining each policy separately, contrary to hypotheses, having spirits and heavy beer sales prohibited at grocery stores (vs. having both permitted) was related to higher odds of low birthweight births (aOR=1.03, 95% CI: 1.01, 1.05) and higher odds of preterm births (aOR=1.03, 95% CI: 1.01, 1.05). Prohibiting spirits sales (and permitting only heavy beer sales) at gas stations (vs. having both spirits and heavy beer sales permitted) was also related to higher odds of low birthweight births (aOR=1.02, 95% CI: 1.01, 1.04), although prohibiting both spirits and heavy beer sales at gas stations was not. Model 4 results also show that prohibiting Sunday off-premise spirits sales (vs. permitting) was related to lower odds of preterm births (aOR=0.98, 95% CI: 0.96, 1.00). Finally, Model 4 results show that having a government monopoly on spirits or on both spirits and wine retail sales (vs. none) were each related to lower odds of low birthweight births (aOR=0.94, 95% CI: 0.93, 0.95; aOR=0.95, 95% CI: 0.93, 0.97 respectively) and lower odds of preterm births (aOR=0.97, 95% CI: 0.94, 0.99; aOR=0.97, 95% CI: 0.95, 1.00 respectively).

**Table 1:** Odds ratios and 95% confidence intervals from models regressing birth outcomes on general population alcohol policies separately, 1972-2019 US Vital Statistics.

	Low Birthweight (N = 160,538,939)^a^				Preterm Births (N = 156,777,912)			
Policy	Model 1^b^	Model 2	Model 3	Model 4^c^	Model 1	Model 2	Model 3^c^	Model 4^c^
Grocery store sales (ref = spirits and heavy beer permitted)								
Heavy beer sales only permitted	1.11 (1.00, 1.23)	1.00 (0.97, 1.03)	1.03 (0.99, 1.07)	1.01 (0.99, 1.03)	1.08 (0.99, 1.18)	0.99 (0.96, 1.03)	1.01 (0.98, 1.04)	1.00 (0.98, 1.02)
Spirits and heavy beer sales prohibited	1.05 (0.95, 1.15)	1.03 (0.99, 1.07)	1.03 (0.99, 1.06)	**1.03** (1.01, 1.05)**	0.99 (0.90, 1.09)	1.03 (0.99, 1.06)	1.02 (0.97, 1.07)	**1.03** (1.01, 1.05)**
Gas station sales (ref = spirits and heavy beer permitted)								
Heavy beer sales only permitted	1.11 (0.99, 1.25)	1.01 (0.98, 1.04)	**1.02** (1.01, 1.04)**	**1.02** (1.01, 1.04)**	1.08 (0.97, 1.20)	0.98 (0.93, 1.04)	0.99 (0.96, 1.02)	1.01 (1.00, 1.03)
Spirits and heavy beer sales prohibited	1.06 (0.94, 1.20)	**0.98** (0.96, 1.00)*	0.99 (0.97, 1.01)	1.01 (0.99, 1.02)	0.98 (0.87, 1.10)	0.97 (0.93, 1.01)	0.97 (0.94, 1.01)	1.00 (0.96, 1.04)
Sunday off-premise spirits sales (ref = permitted)								
Sunday off-premise spirits sales prohibited	1.08 (0.99, 1.18)	1.03 (1.00, 1.07)	1.02 (1.00, 1.04)	1.00 (0.99, 1.01)	1.06 (0.97, 1.16)	1.01 (0.96, 1.05)	0.99 (0.96, 1.02)	**0.98** (0.96, 1.00)*
Blood Alcohol Content limit for driving (ref = none)								
0.10 BAC limit	0.96 (0.91, 1.01)	1.01 (0.97, 1.05)	1.02 (0.98, 1.05)	1.00 (0.99, 1.01)	**1.10** (1.03, 1.18)**	1.00 (0.96, 1.06)	1.01 (0.97, 1.06)	0.99 (0.97, 1.02)
0.05-0.08 BAC limit	0.99 (0.93, 1.06)	1.00 (0.95, 1.04)	1.02 (0.99, 1.06)	1.00 (0.99, 1.01)	**1.20** (1.11, 1.30)***	1.03 (0.97, 1.10)	1.05 (0.99, 1.12)	1.00 (0.97, 1.03)
Government monopoly on spirits/wine retail (ref = none)								
Spirits monopoly only	1.03 (0.91, 1.17)	**0.94** (0.93, 0.96)***	**0.93** (0.91, 0.95)***	**0.94** (0.93, 0.95)***	1.02 (0.91, 1.15)	0.96 (0.92, 1.00)	**0.94** (0.90, 0.98)**	**0.97** (0.94, 0.99)**
Spirits and wine monopoly	0.93 (0.83, 1.04)	**0.89** (0.85, 0.92)***	**0.90** (0.87, 0.94)***	**0.95** (0.93, 0.97)***	**0.86** (0.78, 0.94)**	**0.90** (0.83, 0.98)*	**0.92** (0.87, 0.98)**	**0.97** (0.95, 1.00)*

^a^Ns from Model 4 for government monopoly. Sample sizes vary according to available policy data. Data are collected for gas station policies starting in 1978 (N = 155,110,908 for low birthweight; N = 152,433,735 for preterm births in Model 4).

^b^Model 1 is unadjusted. Model 2 includes fixed effects for state and year. Model 3 further adjusts for maternal age, race-and-ethnicity, marital status, education, and parity, and state-year per capita cigarette consumption, unemployment, and percent living in poverty. Model 4 further adjusts for state-specific linear, quadratic, and cubic trends. All models cluster standard errors by state.

Results from sensitivity analyses: (1) When further adjusting Model 4 for state-level per capital alcohol consumption, Sunday sales is related to preterm births at *P* < 0.066 (aOR=0.98, 95% CI: 0.96, 1.00) and government monopoly on spirits and wine is related to preterm births at *P* < .083 (aOR=0.98, 95% CI: 0.95, 1.00). No other estimates change significance or magnitude. (2) When further adjusting Model 4 for pregnancy-specific policies, no estimates change significance or magnitude. (3) When restricting years to 1989–2019, only government monopoly on spirits and on spirits and wine remain significantly related to low birthweight; no other policy is related to outcomes at *P* < .05.

***P < 0.001, **P < 0.01, *P < 0.05; **Bold** signifies P < 0.05

Table 2 shows results from models regressing birth outcomes on all general population alcohol policies simultaneously. Results follow a similar pattern to models shown in Table 1. In Table 2 fully adjusted models (Model 4), prohibiting spirits and heavy beer sales at grocery stores (vs. permitting both) remained related to higher odds of low birthweight births (aOR=1.03, 95% CI: 1.01, 1.06) and higher odds of preterm births (aOR=1.04, 95% CI: 1.00, 1.08). Prohibiting spirits sales (and only permitting heavy beer sales) at gas stations (vs. permitting both spirits and heavy beer sales) also remained related to higher odds of low birthweight births (aOR=1.02, 95% CI: 1.01, 1.03). Though prohibiting Sunday off-premise spirits sales was no longer significantly related to preterm births in models including policies simultaneously, the magnitude of the relationship remained similar. Having a government monopoly on spirits or on both spirits and wine retail sales (vs. none) were each still related to lower odds of low birthweight births (aOR=0.94, 95% CI: 0.93, 0.95; aOR=0.95, 95% CI: 0.92, 0.98 respectively) but only having a government on spirits sales remained significantly related to lower odds of preterm births (aOR=0.97, 95% CI: 0.95, 1.00). Notably, findings were mostly similar between Models 3 and 4 in Table 2, with only slight differences in magnitudes of effect and precise p-values. For example, while the association between birthweight and prohibiting spirits & heavy beer sales in gas stations was significant at *P* < 0.05 level in Model 3 but not Model 4, point estimates did not differ substantially (0.98 vs. 0.99).

**Table 2:** Odds ratios and 95% confidence intervals from models regressing birth outcomes on general population alcohol policies simultaneously, 1978-2019 US Vital Statistics.

	Low Birthweight (N = 155,023,293)^a^				Preterm Births (N = 152,348,017)			
Policy	Model 1^b^	Model 2	Model 3^c^	Model 4^c^	Model 1	Model 2	Model 3^c^	Model 4^c^
Grocery store sales (ref = spirits and heavy beer permitted)								
Heavy beer sales only permitted	1.07 (0.98, 1.17)	1.02 (0.99, 1.04)	1.03 (0.99, 1.08)	1.01 (0.99, 1.03)	1.04 (0.95, 1.15)	1.01 (0.98, 1.04)	1.02 (0.99, 1.05)	1.00 (0.97, 1.02)
Spirits and heavy beer sales prohibited	1.14 (0.93, 1.39)	**1.07** (1.03, 1.10)***	**1.05** (1.02, 1.09)**	**1.03** (1.01, 1.06)*	1.03 (0.92, 1.16)	**1.06** (1.01, 1.11)*	1.05 (0.98, 1.11)	**1.04** (1.00, 1.07)*
Gas station sales (ref = spirits and heavy beer permitted)								
Heavy beer sales only permitted	1.03 (0.92, 1.15)	1.01 (0.98, 1.04)	**1.02** (1.00, 1.03)*	**1.02** (1.01, 1.03)**	1.03 (0.92, 1.16)	0.98 (0.92, 1.04)	0.98 (0.95, 1.02)	1.01 (1.00, 1.02)
Spirits and heavy beer sales prohibited	1.01 (0.84, 1.22)	**0.96** (0.94, 0.98)***	**0.98** (0.95, 1.00)*	0.99 (0.98, 1.01)	0.95 (0.78, 1.15)	**0.95** (0.92, 0.99)*	**0.96** (0.93, 0.99)*	0.98 (0.95, 1.02)
Sunday off-premise spirits sales (ref = permitted)								
Sunday off-premise spirits sales prohibited	1.06 (0.97, 1.15)	1.03 (0.99, 1.07)	1.02 (1.00, 1.04)	1.01 (0.99, 1.02)	1.07 (0.98, 1.16)	1.00 (0.96, 1.04)	0.99 (0.97, 1.02)	0.98 (0.96, 1.00)
Blood Alcohol Content limit for driving (ref = none)								
0.10 BAC limit	0.97 (0.92, 1.03)	1.01 (0.98, 1.05)	1.02 (0.99, 1.04)	1.00 (0.99, 1.01)	1.07 (1.00, 1.16)	1.01 (0.96, 1.06)	1.01 (0.97, 1.06)	0.99 (0.96, 1.01)
0.05-0.08 BAC limit	1.02 (0.96, 1.08)	1.00 (0.96, 1.05)	1.02 (0.99, 1.05)	1.00 (0.98, 1.01)	1.17 (1.09, 1.26)	1.04 (0.97, 1.11)	1.05 (0.99, 1.12)	0.99 (0.96, 1.02)
Government monopoly on spirits/wine retail (ref = license)								
Spirits monopoly only	1.03 (0.91, 1.16)	**0.95** (0.94, 0.97)***	**0.93** (0.91, 0.95)***	**0.94** (0.93, 0.95)***	1.02 (0.91, 1.14)	0.96 (0.91, 1.01)	**0.94** (0.90, 0.98)**	**0.97** (0.95, 1.00)*
Spirits and wine monopoly	0.90 (0.79, 1.02)	**0.89** (0.86, 0.93)***	**0.90** (0.87, 0.93)***	**0.95** (0.92, 0.98)**	**0.87** (0.77, 0.98)*	0.90 (0.81, 1.00)	**0.91** (0.85, 0.98)*	0.98 (0.94, 1.01)

^a^Ns are from Model 3 for each outcome.

^b^Model 1 is unadjusted. Model 2 includes fixed effects for state and year. Model 3 further adjusts for maternal age, race-and-ethnicity, marital status, education, and parity, and state-year per capita cigarette consumption, unemployment, and percent living in poverty. Model 4 further adjusts for state-specific linear, quadratic, and cubic trends. All models cluster standard errors by state.

^c^Results from sensitivity analyses: (1) When adjusting Model 4 for state-level per capital alcohol consumption, no estimates change significance or magnitude. (2) When further adjusting Model 4 for pregnancy-specific policies, no estimates change significance or magnitude. (3) When restricting years to 1989–2019, only government monopoly on spirits and on spirits and wine remain significantly related to low birthweight; no other policy is related to outcomes at *P* < .05. (4) When excluding gas station sales variable in Model 4 to permit inclusion of data going back to 1972, Sunday sales (aOR=0.98 [0.96, 1.00]) becomes significant (*P* < 0.033) for preterm births; no other results change significance or magnitude.

***P < 0.001, **P < 0.01, *P < 0.05; **Bold** signifies P < 0.05

### Sensitivity analyses

First, when adding per capita alcohol consumption to the fully adjusted separate policy models shown in Table 1 (Model 4), results retained similar magnitudes and levels of significance except Sunday sales was related to preterm births at *P* = .066 rather than *P* < .05 (aOR=0.98, 95% CI: 0.96, 1.00) and government monopoly on spirits and wine was related to preterm births at *P* = .083 (OR=0.98, 95% CI: 0.95, 1.00) rather than *P* < .05. When adding per capita alcohol consumption to the fully adjusted simultaneous policy models (Table 2, Model 4), no estimates changed significance or magnitude. Second, when adding pregnancy-specific policies to Models 4 in Tables 1 and 2, no estimates changed significance or magnitude. Third, when restricting years to 1989–2019 for the fully adjusted separate policy models (Table 1, Model 4) only government monopoly on spirits only and on spirits and wine remained significantly related to low birthweight; no other policies were related to outcomes at *P* < .05. When restricting years to 1989–2019 for the fully adjusted simultaneous policy models (Table 2, Model 4) again only government monopoly on spirits only and on spirits and wine remained significantly related to low birthweight and no other policies were related to outcomes at *P* < .05. Finally, for simultaneous policy models of the 1972–2019 period excluding the gas station policy, results from fully adjusted models (Table 2, Model 4) retained similar magnitudes and levels of significance except the relationship between prohibiting Sunday off-premise spirits sales and preterm births became significant at *P* < .05 (aOR=0.98, 95% CI: 0.96, 1.00; *P* = .033).

## Discussion

### Summary of results

We examined how state-level general population alcohol policies are related to birth outcomes in the U.S. Though we hypothesized that more restrictive policies would be related to decreased odds of low birthweight and preterm births, we found few significant, protective relationships. The most robust protective relationships across model specifications were between government monopolies on spirits retail and low birthweight births. Results were similar when including monopolies on wine. The current results corroborate prior findings showing that government monopolies for spirits are related to decreased drinking among women of reproductive age [21], decreased infant morbidities and maltreatment [22], and improved birth outcomes among young (age 15–24) birthing people [7]. Potential mechanisms by which government monopolies influence alcohol consumption and birth outcomes include that government monopolies on alcohol often limit number of alcohol outlets and hours of days of sales, impose stricter conditions on sales, and increase alcohol prices, all of which are related to reduced drinking [34]. Thus, the protective association between government monopolies on alcohol sales and birth outcomes appears particularly supported.

Results also suggest that prohibiting off-premise spirits sales on Sundays may relate to lower odds of preterm births. Though relationships for Sunday sales were not as robust as for government monopolies, prior research shows that prohibiting Sunday off-premise spirits sales is also related to less overall and less heavy drinking among women of reproductive age [21], which provides evidence of a mechanism for current results. This set of findings corroborates prior studies showing that looser restrictions on Sunday sales are associated with more drinking and worse health outcomes in the U.S. and Canada [35–38],

Contrary to hypotheses, results show that prohibiting spirits and heavy beer sales (vs. permitting both) in grocery stores was related to higher odds of low birthweight births and preterm births; one mechanism that might explain this association is that prohibiting beer and spirits in grocery stores drives people to specialty liquor stores where they might “stock up” and buy (and later consume) more alcohol than if it were available in grocery stores. However, this mechanism has not been examined and should be explored in future studies. Current results also show that prohibiting spirits sales while permitting heavy beer sales (vs. permitting both) in gas stations was also related to higher odds of low birthweight births. While these results are counter-intuitive, they were not robust across all sensitivity analyses and should be interpreted cautiously. Furthermore, multiple other studies from Australia, Sweden, and the U.S. conclude that, among women specifically, higher density of spirits outlets is related to higher odds of drinking, harmful drinking, and drinking problems [39–41]. Similarly, our previous research has found that prohibiting spirits sales in gas stations relates to improved infant morbidities and reduced infant injuries [22]. Thus, the isolated findings specific to birth outcomes do not warrant a shift in interpretations of the broader body of research showing that limiting spirits outlet density is important for health outcomes overall and for women and infants specifically. Still, it might be worth exploring whether restricting all alcohol sales in grocery stores affects where, how much, and what types of alcohol pregnant people purchase.

### Implications for alcohol policy approaches to reduce adverse birth outcomes

Given growing evidence that policies specific to alcohol use among pregnant people do not have their intended benefits and, in some cases, appear counter-productive, policymakers should consider alternative approaches to reduce adverse effects of drinking during pregnancy. In addition to considering repealing or replacing current policies specific to alcohol use among pregnant people, findings from this study indicate that policymakers should consider using general population alcohol policies as alternative approaches to improve outcomes related to drinking during pregnancy instead. In particular, current results support retaining and expanding government monopolies on spirits and wine retail sales. Regarding government monopolies, as of 2024, sixteen states have government monopolies on spirits, and while some states have moved from government monopoly to licensed since Prohibition was repealed in 1933, none have moved from licensed to government monopoly. Thus, despite overwhelming evidence for the public health benefits of government monopolies on alcohol sales [42–48] including for outcomes related to drinking during pregnancy, it is highly unlikely that U.S. states with current license systems will consider moving to government monopolies. Thus, while findings would support expanding government monopolies, practically speaking, findings from these analyses underscore the importance of maintaining the government monopolies that do exist as a strategy for reducing adverse effects of drinking during pregnancy. In addition, while recent policy trends indicate that states are expanding spirits sales to Sundays, our findings also suggest that maintaining or expanding restrictions on Sunday spirits sales similarly could be a promising approach for reducing adverse effects of drinking during pregnancy.

### Strengths and limitations

This is the most comprehensive study of general population alcohol policies and birth outcomes to date, having examined multiple alcohol availability and related policies covering an almost fifty-year period. Most of the period (1985–2019) captures the entire population of singleton births born in the United States and from 1972–1984, some states provided a 100% sample and others a 50% sample. Thus, generalizability and inference are not of concern. Birth outcome data also do not rely on self-report; this is another major advantage over survey data regarding alcohol use, which is often under-reported. Finally, current results are robust across different model specifications and various sets of sensitivity analyses. Though some policies are correlated, e.g., government monopolies and grocery store sales, the similarities across separate and combined policy models suggests that multicollinearity is not an issue when including all policies together in combined models.

Still, results come with caveats. First, birth certificate data are not collected for research purposes and are missing valid data on maternal alcohol and tobacco use [49]. We adjusted for state-level per-capita tobacco consumption and conducted a sensitivity analysis with state-level per capita alcohol consumption instead. Second, states only began to document both race and ethnicity in 1989 and this was phased in across states over the 1990s. Thus, primary analyses did not account for ethnicity, e.g., White Hispanic and White Non-Hispanic mothers are in a single group. Given that birth outcomes are similar between White non-Hispanic and (all) Hispanic births [50], concerns regarding the lack of adjustment for ethnicity should be alleviated. Third, measurement of gestational age, which was used to determine preterm birth, changed over time. To improve consistency across the period, we applied methods to correct for implausible gestational age values in earlier years [51]. Finally, analyses did not adjust potentially relevant general population alcohol policies like state-level taxes and store hours because these data are not available for all states and years analyzed here. Planned analyses will examine alcohol taxes as tax policies are nuanced beyond the scope of current analyses.

### Directions for future research

To establish clear mechanisms for current findings, future research should examine whether general population alcohol policies like government monopolies and restrictions on Sunday sales are related to drinking (e.g., binge drinking, heavy drinking) among pregnant individuals specifically. Given the unexpected finding that prohibiting spirits and heavy beer sales (vs. permitting both) in grocery stores was related to higher odds of adverse birth outcomes, future studies might also examine purchasing patterns and beverage preferences among pregnant individuals. Purchasing patterns and beverage preferences among individuals who may become pregnant and women of reproductive age could also be examined in future studies, given challenges of conducting population-level surveys with sufficient numbers of pregnant individuals. To establish causality, future studies could employ quasi-experimental designs, e.g., difference-in-difference methods that examine individual policies.

## Conclusions

A growing body of research shows that general population alcohol policies might be more effective than pregnancy-specific policies in reducing harms related to drinking among pregnant people. Results show that state government monopolies on spirits and wine retail sales and restrictions on Sunday spirits sales are related to lower odds of low birthweight and preterm births, contributing to the evidence for public health benefits of these policies.
